# Informed consent for research in Borderline Personality Disorder

**DOI:** 10.1186/1472-6939-8-4

**Published:** 2007-05-10

**Authors:** Rachel E Dew

**Affiliations:** 1 Center for Spirituality, Theology, and Health, Duke University Medical Center, Durham, NC, USA; 2 Department of Psychiatry and Behavioral Medicine, Wake Forest University Baptist Medical Center, Winston-Salem, NC, USA

## Abstract

**Background:**

Previous research on informed consent for research in psychiatric patients has centered on disorders that affect comprehension and appreciation of risks. Little has been written about consent to research in those subjects with Borderline Personality Disorder, a prevalent and disabling condition.

**Discussion:**

Despite apparently intact cognition and comprehension of risks, a borderline subject may deliberately choose self-harm in order to fulfill abnormal psychological needs, or due to suicidality. Alternatively, such a subject may refuse enrollment due to transference or the desire to harm him or herself. Such phenomena could be precipitated or prevented by the interpersonal dynamics of the informed consent encounter.

**Summary:**

Caution should be exercised in obtaining informed consent for research from subjects with Borderline Personality Disorder. A literature review and recommendations for future research are discussed.

## Background

Decision-making capacity in psychiatric patients has been the topic of past research [1]. However, psychiatric disorders considered in such research are most often the psychotic and cognitive disorders [2]. This makes sense, as informed consent is traditionally thought to hinge upon comprehension [3].  However, such a viewpoint presupposes a subject that, understanding relative risks and benefits of a proposed action, will usually act in a way that maximizes benefit and minimizes harm to the self. Any deviation from this self-preserving pattern of behavior in the potential research subject is usually thought to result from altruism or such obviously coercive circumstances as financial reward, lack of other access to care, the perception that health care will be withdrawn without participation, etc.

However, what about people that persistently and intentionally harm themselves? A large subpopulation of psychiatric patients suffer pathology which centers around a lifelong tendency to make what appear to others to be bad decisions. They may persistently seek out victim roles and manipulate others to harm them. They may make repeated suicide attempts, or compulsively cut themselves. Can it be considered ethical to draw blood from someone who consented because she has a psychological need to see herself bleed?  Borderline personality disorder (BPD) is a prevalent, chronic, disabling, and treatment-resistant condition. It affects approximately 2% of community dwellers and 20% of psychiatric inpatients [4]. Although randomized clinical trials of both psychotherapeutic and psychopharmacologic treatments for BPD are relatively common [4,5], a literature search on consent issues with BPD subjects reveals little research. Borderlines also likely have poorer general health (a 6.4% rate of BPD has been measured in a primary care population [6]) than the general population and will likely be recruited into non-psychiatric studies, in which the investigators may be unaware of their psychiatric pathology and the issues involved. Although little has been written on issues of research informed consent in borderline personality disorder, I would argue that caution must be exercised in assessing a borderline subject's ability to consent based solely on comprehension of a study.

DSM-IV-TR diagnostic criteria for borderline personality disorder stipulates that a patient display five of the following nine features [7]:

1) frantic efforts to avoid real or imagined abandonment

2) a pattern of unstable and intense interpersonal relationships characterized by alternating between extremes of idealization and devaluation

3) identity disturbance: markedly and persistently unstable self-image or sense of self

4) impulsivity in at least two areas that are potentially self-damaging (e.g., spending, sex, substance abuse, reckless driving, binge eating)

5) recurrent suicidal behavior, gestures, or threats, or self-mutilating behavior

6) affective instability due to a marked reactivity of mood

7) chronic feelings of emptiness

8) inappropriate, intense anger or difficulty controlling anger

9) transient, stress-related paranoid ideation or severe dissociative symptoms

Common clinical features include frequent intense mood swings, the inability to be alone nor to tolerate intimacy, extreme dependency on others alternating with sudden hostility, perceiving others as all good or all bad ("splitting"), chronic self-mutilation (often described as relieving emotional pain), and chronic suicidality. BPD is frequently comorbid with substance abuse, depression, anxiety, and eating disorders [8]. Any of these symptoms could have implications for the informed consent process.

## Discussion

The Belmont Report, the Nurembug Code, and the Declaration of Helsinki all discuss the need for informed, voluntary consent from research subjects.  Possible issues concerning the validity of informed consent in borderline subjects center on the voluntariness of consent. Most borderline patients, except if in a brief psychotic episode when under severe stress, have normal cognitive function, and will be able to comprehend and speak about the risks and benefits of a study.

However, long-term health benefits or more abstract service to society may not be as important to them as their immediate psychological needs. Such needs may include recapitulations of childhood abuse. For example, they may involve a need to feel the study doctor is hurting them or putting them in danger, and the subject may consent to research in order to gain this position. BPD subjects may have an immediate need to use the idealizing side of the splitting defense; they may feel special because they were selected for the study, and consent in order to have more contact with the idealized doctor. Alternatively, a borderline subject's actions may be motivated by the abrupt devaluing tendency seen in their interpersonal relationships: they may refuse to consent to a study that could significantly benefit them based on unwarranted anger toward the study staff. Finally, a borderline patient may enter a study because she is suicidal, and hopes he or she will die as part of the study.

Just as financial compensation may be in fact coercive to some populations, these perceived benefits may in fact be considered coercive for the borderline subject. The Freudian concept of psychic determinism, which states that all decisions are predestined by the interplay of intrapsychic forces and therefore always necessarily subjective, might in the extreme lead us to conclude that no consent is purely voluntary. However, the concern that decisions are not made based on logical consideration but rather on psychological needs applies especially to borderline subjects, for whom rational decision making is a clear weakness.

Carl Elliott raised somewhat similar concerns regarding informed consent for research with the severely depressed [3]. Recognizing that in an extremely depressed state, a subject may understand risks but not be concerned about them, Elliott called for an appreciation of emotional and motivational factors in the informed consent process. He argued that consent might not be considered valid for a depressed subject because of state-dependent changes in priorities which wouldn't coincide with the subject's "usual" personality. This argument flounders when we consider the borderline subject, whose most prominent personality trait is instability. Elliott also contends that consent may be considered invalid if it is not motivated in part by self-interest. This thesis applies better to BPD subjects, who may enroll hoping to be harmed. It does not, however, completely acknowledge that BPD patients have wants and needs that they try to meet; these are just wants and needs that are strange to those without BPD.

In light of these concerns, should borderlines be excluded from research studies? Obviously not. First of all, this would not be practically possible with current standards. There are many more patients with BPD than those who have it documented in their medical records. Even if a study involves formal psychiatric evaluation, practitioners often mistake BPD for other conditions (for example, chronic major depression or bipolar disorder) [6]. This may be due to lack of long-term knowledge of the subject and his or her persistent coping mechanisms. Alternatively, practitioners hesitate to assign a diagnosis that many consider pejorative and untreatable.

Even if borderlines could be more reliably identified prior to informed consent, they cannot be ethically excluded from research because this condition desperately needs better treatments [4,5]. While there is some evidence of efficacy for antidepressants, antipsychotics, Dialectical Behavior Therapy, and other psychotherapies, recent systematic reviews on both psychopharmacology and psychosocial interventions for BPD called for more research. What specialized treatments there are, are not widely available, and many borderlines are prescribed somewhat haphazard polypharmacy regimens with little empiric basis.

If borderline subjects' chronic problems with transference, splitting, impulsivity, paranoia, self-injury, and suicidality complicate the informed consent process, but if this process is none the less imperative, how are we to proceed?  As very little research has been done in this area, hard guidelines for informed consent are premature. But future research should seek to address such questions about informed consent. It may be that borderlines should undergo the consent process with people who are used to working with this disorder. This could include experienced mental health nursing staff, or, more likely, someone with psychotherapy training. Experience and training in psychotherapy enables one to monitor transference and counter-transference throughout the encounter with the borderline subject, lending insight into the subject's current motivational state and helping keep her or his affect from spinning out of control. These skills may also minimize unconscious acting out by the study team. For example, someone inexperienced with borderlines may not recognize that he or she is being idealized and may think "we really will be able to do great things for this person; I must get her into the trial." Or an inexperienced clinician may encounter a hostile subject and unconsciously adjust entry criteria such that she does not qualify, or over-emphasize risks in a way that leads her to decline enrollment. Future studies should aim to determine whether psychotherapeutic knowledge and experience affects the informed consent process with BPD subjects. Experiments could potentially involve having researchers with varying levels of psychotherapeutic experience consent the same subjects for similar studies, comparing the resultant interpersonal interactions.

Another relevant question is whether the person obtaining consent can ethically be the subject's therapist or psychiatrist. One of the criteria for the diagnosis of BPD is "frantic efforts to avoid real or imagined abandonment."  Whatever the consent form says about lack of consent not affecting provision of medical care, borderlines live in a world where desertion is always seconds away. Thus, they may be easily coerced to participate based on the fear of loss of love; they also might refuse a trial that could benefit them, in order to test the loyalty of those seeking their participation. This question could be tested by comparing a consent experience using a BPD's own therapist with another using an unknown researcher.

In addition, given the chronic impulsivity BPD subjects display, it may be that informed consent processes that take place over more than one visit could increase the validity of the consent (as well as help retention rates). Letting the subject consider the study away from the interpersonal context of the clinic may help him to be more objective about his desires and less interpersonally reactive.  Multiple consents could be fairly easily added to a research protocol with follow-up subject satisfaction surveys and comparisons of retention rates.

Whether proxy decision-makers apply to this situation is another question.  Severely-ill borderlines may have court-appointed guardians that will be enlisted in the consent process; however, in some cases in which the patient is his or her own guardian but seems incapable of making a rational decision, surrogate decision-makers could be considered.

## Summary

Clinical research on borderline personality disorder is alarmingly underdeveloped considering the prevalence and costliness of this disease [4,5].  This problem is likely in part related to practical difficulties involved in safely recruiting and treating borderline research subjects. Research in this area must be done. Nonetheless, due to the questions of motivation and voluntariness, caution must be exercised in gaining informed consent from borderline subjects.

## Competing interests

The author(s) declare that they have no competing interests.

## Authors' contributions

All work on this manuscript was performed by RD.

## Pre-publication history

The pre-publication history for this paper can be accessed here:


